# Proteoglycan degradation mimics static compression by altering the natural gradients in fibrillar organisation in cartilage

**DOI:** 10.1016/j.actbio.2019.07.055

**Published:** 2019-10-01

**Authors:** Sheetal R. Inamdar, Ettore Barbieri, Nicholas J. Terrill, Martin M. Knight, Himadri S. Gupta

**Affiliations:** a Institute of Bioengineering and School of Engineering and Material Science, Queen Mary University of London, London E1 4NS, United Kingdom; b Center for Mathematical Science and Advanced Technology, Research Institute for Value-Added Information Generation, Japan Agency for Marine-Earth Science and Technology, 3173-25, Showa-machi, Kanazawa-ku, Yokohama-city, Japan; c Harwell Science and Innovation Campus, Diamond Light Source, Harwell, Didcot OX11 0DE, United Kingdom

**Keywords:** Cartilage, Biomechanics, Nanostructure, Collagen mechanics, Structural gradients

## Abstract

Structural and associated biomechanical gradients within biological tissues are important for tissue functionality and preventing damaging interfacial stress concentrations. Articular cartilage possesses an inhomogeneous structure throughout its thickness, driving the associated variation in the biomechanical strain profile within the tissue under physiological compressive loading. However, little is known experimentally about the nanostructural mechanical role of the collagen fibrils and how this varies with depth. Utilising a high-brilliance synchrotron X-ray source, we have measured the depth-wise nanostructural parameters of the collagen network in terms of the periodic fibrillar banding (D-period) and associated parameters. We show that there is a depth dependent variation in D-period reflecting the pre-strain and concurrent with changes in the level of intrafibrillar order. Further, prolonged static compression leads to fibrillar changes mirroring those caused by removal of extrafibrillar proteoglycans (as may occur in aging or disease). We suggest that fibrillar D-period is a sensitive indicator of localised changes to the mechanical environment at the nanoscale in soft connective tissues.

**Statement of Significance:**

Collagen plays a significant role in both the structural and mechanical integrity of articular cartilage, allowing the tissue to withstand highly repetitive loading. However, the fibrillar mechanics of the collagen network in cartilage are not clear. Here we find that cartilage has a spatial gradient in the nanostructural collagen fibril pre-strain, with an increase in the fibrillar pre-strain with depth. Further, the fibrillar gradient changes similarly under compression when compared to an enzymatically degraded tissue which mimics age-related changes. Given that the fibrils potentially have a finite capacity to mechanically respond and alter their configuration, these findings are significant in understanding how collagen may alter in structure and gradient in diseased cartilage, and in informing the design of cartilage replacements.

## Graphical abstract

**Figure:**
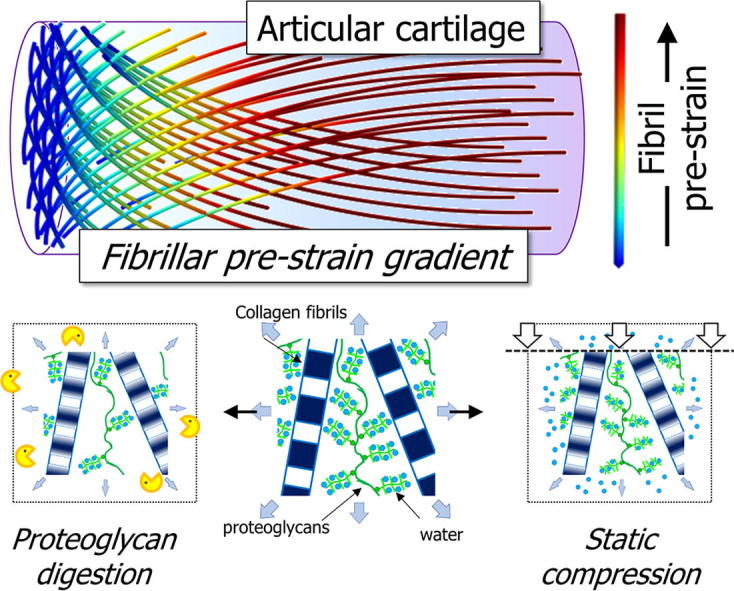

## 1 Introduction

Many connective tissues are found to possess a structural gradient in terms of their composition and architecture in order to fulfil their functional role [1]. Such gradients are found both at the interfaces between different tissue types, as well as – at different length scales – within individual tissues. For example, the interface between tendon and bone is crucial for the anchorage of muscle, whilst bone itself possesses a three dimensional structure (lamellar osteons) that are graded by the varying orientations of fibrils and mineral platelets in 3D [2], [3]. As a result, such tissues often have complex, multi-phase and spatially varying interactions between the components that comprise the hierarchical structure. In particular, articular cartilage (AC) is a case where both types of gradients can be observed [4]. The primary role of AC is to provide a smooth lubricated surface between contacting bones, which allows both frictionless sliding as well as a reduction in contact stresses to the underlying bone [5]. The depth-dependent variation in structure in AC is believed to be necessary for its mechanical role of a sliding on one side and firm anchorage to bone on the other whilst reducing the interfacial shear stresses between joint surfaces [6]. However, whilst the tissue- and micro-level architectural gradients in AC are well understood [7], the corresponding spatial variation in the nanoscale and molecular structural parameters are much less investigated. Understanding these ultrastructural gradients and their biomechanical significance will be critical in developing new biomaterials for articular cartilage repair and replacement.

**Fig. 1:**
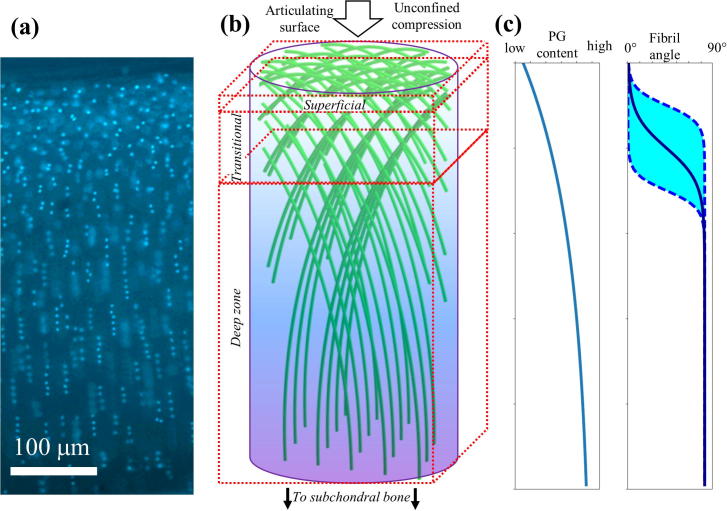
(a) Epifluorescence microscopy of a full thickness cartilage core-section; cell nuclei labelled with DAPI are visible as light dots with (b) The Benninghof [15], [16] arcade-like sheets of collagen fibrils (green; colour online) in a proteoglycan matrix (blue/violet shade; colour online), showing the change in fibril orientation from parallel to the joint surface in the superficial zone, through an intermediate transitional zone with multiple orientations down to a deep zone where fibrils are oriented perpendicular to joint surface; arrow indicated direction of compression (c) Schematics of known microstructural and compositional variation in AC: left: proteoglycan concentration increases [12] from superficial to deep zone and right: fibril angle changes from parallel to perpendicular to joint surface. The central solid line denotes the varying direction of a particular group of fibrils (of the type depicted in Fig. 1(b)), and the shaded region are meant to schematically indicate the wide-range of projected angles in the transitional zone, rather than the absolute values of the angles. (For interpretation of the references to colour in this figure legend, the reader is referred to the web version of this article.)

Like all biological connective tissues, cartilage has a hierarchical architecture from the nanoscale, through the micro- and tissue-level [8]. In particular, at the nanoscale, cartilage is comprised of predominantly type II collagen fibrillar network restraining the swelling of a phase of negatively charged, highly hydrated proteoglycan molecules [9]. It is believed that the two components alongside the associated water form a pre-stressed fibrous hydrogel-type structure that has the capacity to withstand high and repetitive compressive loading [10]. At the microscale, however, the tissue as a whole can be split into a further three zones, i.e. superficial (10–20%), transitional (40–60%) and deep (30–40%) [8]. As summarized in Fig. 1, both the collagen II fibrils and the proteoglycans vary in their content across the zones; both increasing in content with depth such that the largest quantity of each is found in the deep zone [11], [12]. Furthermore, the collagen type II fibrils also vary in their architecture in the different zones such that they are predominantly aligned parallel to the surface in the superficial, in a range of orientations in the transitional zone, and perpendicular to the surface in the deep zone [13], [14], [15], [16]. The particular changes in orientation in articular cartilage is related to the specific resistance to loading at different parts of the tissue, whereby fibrils in the superficial zone are able to deal with shear forces whilst the deeper zone fibrils transition to a 90° shift to allow the tissue to anchor into the adjacent bone surface by fibrils which insert vertically into the interface [17], [18]. This gradient in structure and composition is highly effective in minimising the stresses transduced from the surface of cartilage through to the underlying bone [19]. Recently, the collagen fibrils have been shown to play a crucial role in resisting compression through fluctuations in the existing pre-strain amongst the fibrils [20]. Alongside this, a study utilising atomic force microscopy methods to determine the tensile elasticity of collagen fibrils showed that osmotic pressure influences the mechanical response of the fibrils [21].

However, in musculoskeletal degeneration, ageing and conditions like osteoarthritis (OA), the overall functioning of the joint is disrupted, accompanied by structural and compositional changes such as the thinning of the cartilage layer and loss of proteoglycans [22], [23], [24]. Further, this leads to changes in the local mechanobiological environment and specific changes to the distribution and compositional gradients – such as changes to the fibrillar orientation [14]. Such changes have been hypothesized to be influential in driving age-related dysfunction and osteoarthritis [25]. Computational models, treating cartilage as a fibril-reinforced poroviscoelastic material, have previously been developed, linking changes in proteoglycan concentration to swelling pressure and collagen prestress [26], [27]. Nevertheless, there is limited experimental evidence quantifying such changes, especially at the nanoscale [14]. To understand how the spatially resolved nanoscale architecture changes in cartilage due to loading or matrix degradation, small-angle X-ray scattering (SAXS), particularly with a synchrotron microfocus beam, is a promising approach. SAXS has been widely used to quantify the nanostructural changes in the collagen architecture of biological tissues [28], [29], [30], [31]. More specifically, previous groups have quantified both spatially resolved fibrillar orientation [32], [33] as well as nanostructural parameters such as the D-periodicity and inter-fibrillar spacing in biological tissues such as bone and tendon [34], [35], [36], [37]. However there have been far fewer studies utilising X-ray diffraction in determining such parameters in cartilage [20], [38]. Our previous work [20] showed that SAXS could pick up transient changes in fibrillar pre-strain in the deep zone of AC during macroscopic stress-relaxation. However, we did not investigate the interplay between spatial gradients in AC-structure and levels of fibrillar pre-strain across the tissue, nor how externally applied loads and matrix modification – as occurs, for example, in musculoskeletal degeneration – could potentially alter the nanoscale mechanical equilibrium between fibrils and proteoglycans. Here we utilise SAXS to measure the depth-wise gradient in fibrillar architecture in terms of the D-periodicity and associated ordering in full thickness bovine cartilage, and measure the spatially resolved nanostructural effects of mechanical loading and enzymatic degradation. Using previously published models of cartilage ultrastructure as a fibril-matrix composite [26], [27], we link these experimental changes in fibrillar structure to alterations in the local mechanical environment and swelling pressure.

## 2 Material and methods

### 2.1 Sample collection and enzymatic degradation

Cartilage explants were taken from the metacarpal-phalangeal joint of freshly slaughtered adult bovine steers (aged 18–24 months) which were obtained from a local abattoir. This model using cartilage from the bovine metacarpal-phalangeal joint is well-characterised and provides a readily available homogenous group of samples suitable for mechanical compression testing and without the inherent variability associated with human cartilage samples. The joints were washed and soaked in both biological detergent and 70% ethanol prior to opening of the joint. This is a routine procedure to reduce the risks of contamination in subsequent cell and tissue culture. Since the joint is not opened at this stage the cartilage tissue does not experience any effects of the ethanol soaking. Full thickness cartilage explants were removed using 2 mm biopsy punches and a scalpel from the central load bearing areas of the proximal surface of the joint. The joint surface was kept hydrated using phosphate buffered saline solution (PBS) (Sigma-Aldrich, Poole, UK) during the extraction process. The explants were then transferred into a 96-well plate and left to rest for 24 h at 37 °C. Following this, half the samples were incubated in buffer solution whilst the other half were treated with chondroitinase ABC at 0.1U/ml (Sigma-Aldrich, Poole, UK) using methods previously described [20]. Lab-tests to test for sGAG release were carried out on a parallel batch of samples, using a calibrated 1,9-dimethylmethylene blue (DMMB) complexation assay (Supplementary Information Experimental–Section 1, Table S1 and Figs S2-3). Following incubation for 24 h, the samples were then transferred into Eppendorf tubes with PBS and snap frozen in liquid nitrogen for storage until the experiments were conducted at Diamond Light Source.

### 2.2 Protocol for in-situ mechanical testing combined with SAXS

All samples were tested at beamline I22 at Diamond Light Source (Research Campus Harwell, Harwell). The beam-size was measured to be ∼15 µm with an energy of 14KeV. In a manner similar to that described by us earlier [20], SAXS patterns were taken with a Pilatus P3-2M detector (Dectris, Villingen, CH). A silver behenate (AgBe) calibration standard was used to obtain the sample to detector distance of 841.7 ± 1.0 mm. An exposure time of 0.5 s were used per SAXS pattern. Incident beam intensity was reduced via a 0.05 mm Molybdenum attenuator. The accessible wavevector range relevant for meridional SAXS was from 0.2 nm^−1^ to 0.9 nm^−1^ – due to low-angle parasitic scattering near the beam-stop in some locations (especially close to the edge of the platens at either end of the cartilage), the 3rd order meridional peak at ∼0.27–0.28 nm^−1^ was occasionally affected, and hence the clearest peak (5th order) was used for analysis as described below. For the *in situ* SAXS experiments, the samples were thawed out and equilibrated at room temperature and then placed in line with the beam onto a customised holder on a micro-compression tester that was mounted at the beamline. Within the unconfined sample holder, a tare load of 0.1 N was first applied to ensure contact between the loading platen and sample surface. Using a relative scan, an initial vertical line scan was performed through the full thickness of the tissue in the uncompressed state, with a vertical scan step of 20 μm. Samples were then compressed to 20% strain level at a rate of 20%/min and then held for a period of 15 min to allow complete relaxation of the tissue prior to the secondary line scan through the full thickness. All samples were maintained in a hydrated state with phosphate buffered saline solution.

When carrying out scanning SAXS on samples where the depth along the beam (here 2 mm) is much larger than the beam size (15 µm) and step-size (20 µm), cross-talk and projection effects and associated errors need to be considered. As samples are, by nature, disc-like with variation along the thickness (here ∼500 µm), possible tilting of the sample with respect to the horizontal base-table or with respect to the beam could lead to cross-talk effects, where the beam enters a particular zone of tissue at one end but exits at a slightly displaced point (See Supplementary Fig. S1). The use of a tare-load of 0.1 N to obtain a pre-load contact of the sample with respect to the loading platens will eliminate the first effect. To estimate the crosstalk error with possible beam tilt, from the X-ray calibration with silver behenate, the X-ray beam tilt is <0.1°. Hence the total vertical “drift” of the beam will be 2000 µm × sin (0.1°) ∼3.5 µm, i.e. around 5 µm. Therefore, the SAXS structural information should be considered as averaged over a vertical zone of (20 + 5) μm = 25 μm, rather than 20 μm, leading to an error in fractional position (relative to sample thickness) of ∼1%.

### 2.3 SAXS analysis

The parameters described below were calculated from the SAXS patterns using methods partially described previously [20].

#### 2.3.1 Collagen D-Periodicity

**Fig. 2:**
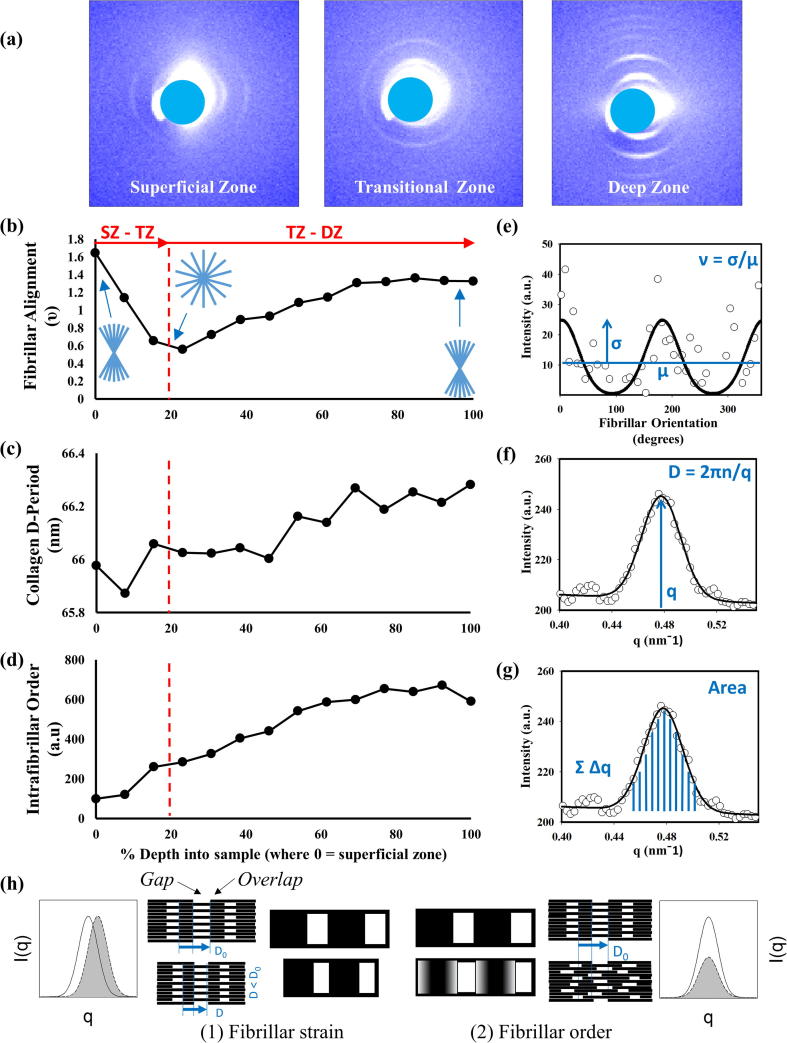
The depth-wise variation in SAXS derived parameters in bovine articular cartilage. Sample SAXS patterns from a single sample of full thickness cartilage are shown in part (a) (beam-stop region masked with a circle). The depth-wise fibrillar alignment (ν-parameter), collagen D-Period and intrafibrillar order versus depth are shown in (b)–(d) respectively. In (b), blue line-patterns show different degrees of fibrillar alignment across the tissue and the correlation with ν-parameter. A representative radial integration is shown in (e) which indicates how the ν-parameter is calculated which a representative azimuthal integration is shown in (f) and (g) showing how both D-period and intrafibrillar order are determined, respectively [40]. (h) Schematic showing the structural meaning of fibrillar strain and order, in terms of inter-molecular arrangement within the fibril and related SAXS pattern. The staggered arrangement of tropocollagen molecules is shown schematically in the centre, with the regions of high density (overlap) and low density (gap) shown at the fibrillar (centre) and intrafibrillar level (offset centre). The left images show fibrils undergoing compressive strain (shift in D-period) without intrafibrillar rearrangement (shift in meridional SAXS peak). The right image show intrafibrillar rearrangements of molecules (disordering), leading to reduction of SAXS peak intensity. As the data shown is from a single sample, no error bars are indicated on the plots. (For interpretation of the references to colour in this figure legend, the reader is referred to the web version of this article.)

The fibrillar D-period was measured from each SAXS pattern by first integrating the 2D SAXS intensity azimuthally from 0° to 360° around the 5th order meridional peak, utilising 100 bins, which resulted in an intensity profile referred to as *I*(*q*). The SAXS meridional diffraction peak arises from periodic fluctuations of electron density along the collagen fibril axis, with dense (overlap) zones alternating with less dense (gap) zones due to a staggered arrangement of intrafibrillar tropocollagen molecules (shown schematically in Fig. 2(h), left). To calculate the SAXS intensity coming from the meridional Bragg peak alone, the diffuse SAXS background around the 5th order meridional peak (0.40–0.55 nm^−1^) was subtracted by including a linear background term together with a Gaussian in the fit function, similar to our earlier work on bone and cartilage and echinoderm collagen [20], [39], [40]. From the fit, the peak position is used to calculate the value of D-period where the position (*q*_0_) is equal to 2π/D. This was run across all the samples using customised Perl scripts that were integrated with Gnuplot software (www.gnuplot.info) and Fit2D [41].

#### 2.3.2 Intrafibrillar order

The *I*(*q*) profile, more specifically the area under the Gaussian peak profile and thus intensity, provides an estimate of the intrafibrillar disordering observed amongst the fibrils. Given that each SAXS pattern provides intensity that corresponds to the average signal from the sampling volume, this parameter provides an estimate of the average level of intrafibrillar disordering amongst the group of fibrils. Intrafibrillar disordering refers to the blurring of the gap-overlap interface (Fig. 2(h), right) due to axial displacements of tropocollagen molecules, and lead to an decrease in meridional peak intensity via a Debye-Waller term [20], [42]. The higher the intensity the more ordered the intrafibrillar structure is and therefore a lower intensity represents a higher level of disordering at the molecular level. Peak intensity was taken from the area under the unfitted *I*(*q*) profiles from the 5th order meridional peak. As the beamline was operating in top-up mode, the incident X-ray beam intensity is nearly constant, with small fluctuations around 1–2% of the average value and no long-term secular trend (See Supplementary Fig. S7 for a representative example of incident beam intensity over the course of a line-scan). Therefore no additional normalisation with respect to incident beam intensity was carried out.

#### 2.3.3 Fibrillar alignment

The fibrillar alignment is determined using the ν-parameter [40] which measures the angular anisotropy in *I*(χ) as a function of azimuthal scattering angle χ. As shown in Fig. 2c, a radial integration is performed in a narrow radial band, to average intensity around the SAXS collagen peak to produce an intensity profile *I*(χ). Within the small-angle (SAXS) approximation (i.e., neglecting the curvature of the Ewald sphere), the intensity of *I*(χ) at a particular χ is proportional to the number of fibrils within the sample volume oriented at χ in a 2D plane normal to the beam. A 0-360° cake sector was applied to the 5th order peak, utilising 100 bins in the azimuthal direction, to generate the intensity profiles. The ν-parameter is calculated by dividing the standard deviation of I(χ) by the mean of *I*(χ) [40]. The more aligned the fibrils are (or more angularly anisotropic), the larger is the standard deviation in *I*(χ) away from the mean and thus the ν-parameter increases. Conversely, the wider the distribution (or more angularly isotropic), the lower the value in ν and thus increased fibrillar disorder in terms of alignment [40]. Schematics of aligned and less-aligned fibrils are shown in Fig. 2(b).

#### 2.3.4 Radiation damage

We tested for radiation damage by carrying out SAXS measurements with progressively increasing exposure times (0.1 s to 5 s) and obtained a linearly increasing trend for SAXS peak intensity with no saturation observed (Supplementary Fig. S5). Hence, our configuration (for the SAXS measurements above) of measuring the same or adjacent tissue for 2 × 0.5 s (well below 5 s) are not influenced by structural damage due to radiation.

### 2.4 Statistical analysis

Sample state (SS) from the above protocol can be classified into three groups: CTR (control, untreated cores, before loading), CPL (control, untreated cores, upon loading to 20% strain) and CHD (chondroitinase-treated cores, not loaded). From the above, the nanoscale (SAXS-derived) parameters D and intrafibrillar order (IFO) are obtained as a function of the experimental variables. As the experimental variable include both discrete factors describing the sample state SS (unloaded control CTR, loaded control CPL, and chondroitinase-treated CHD) and continuous factors (position along the sample thickness, which is not identical across samples due to inter-sample thickness variability), a generalized linear model approach was used in the statistical package R (www.r-project.org), where trends with the percentage sample thickness *x* (a continuous variable) and with the discrete factor levels (CTR, CPL and CHD) are tested. Percentage sample thickness *x* is 0 at the superficial side of the sample and increased to 100 on going toward the deep zone of the cartilage core. Linear models of the type D (or IFO) ∼ *x* + SS + *x*:SS were used. ANOVA using Type III sums of squares (to include the effect of the continuous covariate *x*) was applied to the linear model to test for significant variations (*p* < 0.05). In the three-term linear model above, the first term tests for a significant variation of D with *x*, the second term on whether the *x* = 0 (intercept) values for D differ (i.e. at the superficial zone), and the third term on whether the gradients of the lines for the different SS levels differ significantly. Summaries of the linear model results and statistical tests, and graphical depictions of the fitted regression lines and confidence intervals, are reported in the Results. Normality is checked via Shapiro-Wilk tests on residuals.

#### 2.4.1 Sample number and pooling

Four samples (G1-G4) were tested in the CTR/CPL group, and three samples (CHD1-3) in the CHD group. G1-CPL data is not available due to a failure of the mechanical test software after loading G1. Hence, data for CTR was obtained by pooling G1, G2, G3 and G4, for CPL by pooling G2, G3 and G4, and for CHD by pooling CHD1, CHD2, and CHD3. Bins of 10% width were chosen in *x*. To avoid biasing the groups toward a particular (thicker) sample due to possibly multiple points in a bin for thicker samples, only the point closest to the mid-point of each bin was taken for each sample (i.e. equal weighting across samples). For the line scan of each sample, the first and last point at which a SAXS signal from cartilage was observed were taken as *x* = 0 and *x* = 100 respectively. For *N* sample scan-points, *x* is calculated for the *i*^th^ point as 100(*i*/*N*), 1 ≤ *x* ≤ *N*. Due to scattering from the loading platens leading to streaks interfering with the SAXS patterns at *x* = 0 and 100, these boundary value points were not included in the regressions.

### 2.5 Mechanical modelling

A fibril-matrix model of the cartilage ultrastructure, where a representative volume element of the ECM (at the scale of a few microns) contains principally of Type II collagen fibrils and hydrated proteoglycans, can be developed from existing models [26], [27], [43], [44]. The net stress in the volume element is the combination of a swelling pressure Π, and the reaction stress σ_F_ from the extension of the fibrillar network (which can bear only tensional loads) and the compression of the matrix σ_M_. Assuming isotropic deformation and material properties, it is possible to obtain an expression for the swelling pressure Π

$$\Delta \Pi =\left(1-\phi_{F}\right)\frac{2}{\lambda^{3}}\left[C_{1}\left(\lambda^{2}-1\right)+12D_{2}\log\lambda \right]+\phi_{F}\frac{2}{3}4\pi C_{4}\beta \frac{{\left(\lambda^{2}-1\right)}^{\beta -1}}{\lambda }$$

where λ is the fibril stretch ratio (1 + strain), *ϕ*_F_ = 0.15 is the fibril volume fraction [45], and the material parameters *C*_1_ = 4.3 × 10^−3^ MPa, *D*_2_ = 2.7 × 10^−4^ MPa, *C*_4_ = 2.0 MPa and β = 2.5 are taken from [26]. Details of the derivation are provided in Supplementary Information. From this expression, the changes in strain in the fibrillar network (λ−1) can be linked to the swelling pressure Π, with increased values of Π corresponding to increased values of fibrillar D-period (corresponding to increased fibrillar pre-strain).

## 3 Results

### 3.1 Depth-dependent fibrillar SAXS parameters in bovine articular cartilage

To demonstrate the spatially-resolved change in SAXS patterns across AC, a representative scan is shown in Fig. 2. Fig. 2a shows qualitative differences in the intensity and anisotropy of the peaks in the 2D SAXS patterns, indicative of the variation of structure between the superficial (left), transitional (middle) and deep (right) zones. The azimuthal (angular) position of the meridional peaks signpost the orientation of the fibrils relative to the surface of the tissue. In the superficial zone the peaks are found on either side of the beam centre, indicating the fibrils lie parallel to the tissue surface, whilst in the deep zone the peaks are shifted by around 90°, with the fibrils perpendicular to the surface of the tissue. In contrast, the Bragg peaks in the SAXS pattern of the transitional zone appears as full rings with peak intensity at all azimuthal angles. Full-range *I*(*q*) profiles for these 2D SAXS images are presented in Supplementary Information, Fig. S6.

In order to quantitatively establish these differences in the SAXS profiles in a spatially-resolved manner, we consider the variation in a) fibrillar alignment b) fibrillar D-period and c) intrafibrillar order as deduced from total SAXS peak intensity as described in the Material and Methods (SAXS Analysis). For a), a representative sample trace of the ν-parameter (indicating fibrillar alignment) is shown in Fig. 2b. We can see that the degree of orientation is high in the superficial zone, reduces smoothly to a minimum in the transitional zone, and then – again smoothly, with no discontinuous transition – increases the further one gets into the deep zone. Secondly, we also observe a clear increasing trend in the fibrillar D-period on going from the superficial cartilage to the deep zone. Fig. 2c shows that while at the surface of the tissue the D-period is ∼66 nm and towards the deep zone the value increases to ∼66.4 nm marking a 0.6% increase overall. This increase in D-period is equivalent to a 20% change in the total collagen pre-strain as a result of hydration when assuming dry collagen to have a D-period of 65 nm versus 67 nm in fully hydrated fibrils. Furthermore, there is also a similar trend in the total 5th order peak intensity and this intrafibrillar order, as shown by Fig. 2d, where the intensity is ∼7 times larger in the deep zone when compared to the superficial zone.

### 3.2 Effects of loading on depth-wise fibrillar D-period and peak intensity

**Fig. 3:**
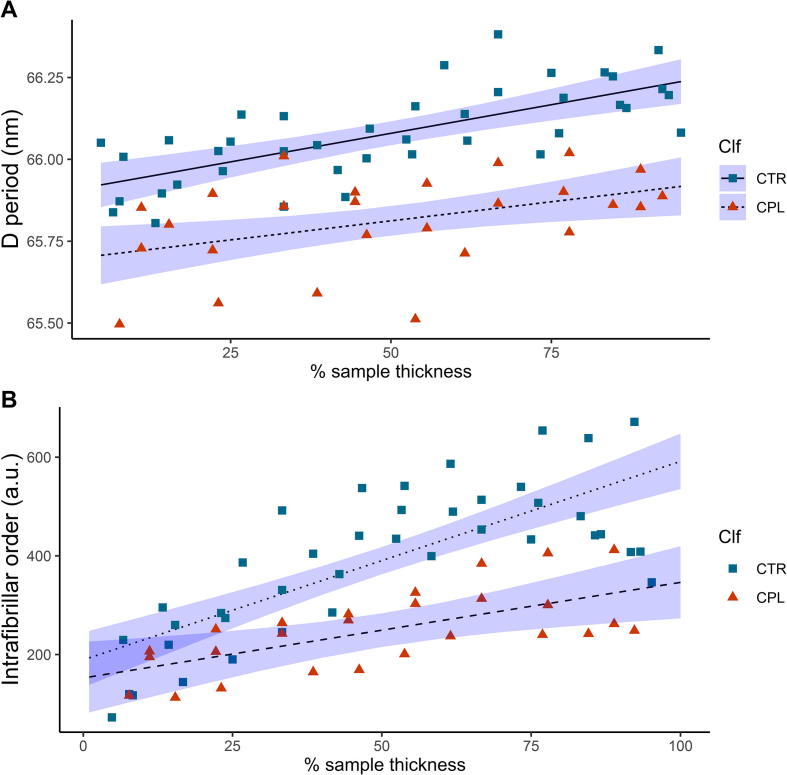
Changes to the depth-wise collagen D-period (a) and associated intrafibrillar order (b) following 20%/min static compression to 20% strain level, where the squares represent values prior to loading (Sample State (SS) = CTR) and the triangles represent post compression and relaxation (SS = CPL). Solid (CTR) and dashed (CPL) lines denote results of the linear regression models: D ∼ X + SS + X:SS (for (a)) and Int ∼ X + SS + X:SS (for (b)). Shaded regions denote confidence intervals on the linear regressions. Linear regression and statistical parameters reported in Table 1.

Progressing from the observation of trends in an individual sample (Fig. 2), we report variation of SAXS parameters pooled across samples and plotted as a function of % thickness in to sample *x* and compared the unloaded (CTR) and loaded (CPL) cases. Fig. 3a shows these parameters together with the linear regressions and confidence intervals, comparing zero load versus static compression of 20% tissue strain. Comparing CTR vs CPL, it appears that the regression lines have similar slopes, but the CPL-line is shifted to overall lower D-period compared to CTR. This qualitative impression is confirmed from the linear model fit and ANOVA results (Table 1 A(i)), where it is seen that a) there is an extremely significant (*p* < 0.001) change of D period with *x* for CTR b) a very significant (*p* < 0.01) reduction in the D-period intercept (at *x* = 0) for CPL compared to CTR and c) no significant difference between the slopes (*p* = 0.24).

**Table 1:** Variation of D-period and SAXS intensity with depth, loading condition and enzymatic treatment: Summary of linear model and ANOVA test results for A) unloaded, control cartilage cores (Sample State (SS): CTR) vs loaded (and held at 20% strain) control cores (SS: CPL) and B) unloaded, control cartilage cores (SS: CTR) vs chondroitinase-treated cores (SS: CHD). i) and ii) denote model results for D-period and SAXS intensity in each case. Estimated parameters and errors, t- and F-statistics and *p*-values for different terms are shown, along with adjusted *R*^2^ and Shapiro-Wilk normality tests (*p*(SW)). Not significant (ns): *p* > 0.05; significant (*): *p* < 0.05; very significant (**): *p* < 0.01; extremely significant (***): *p* < 0.001. For both A) and B), the linear regressions for D versus *x* are significantly (CTR-CHD) and extremely significantly (CTR-CPL) shifted relative to CTR (*p*-value of SS-term), while slopes are similar (*p* > 0.05 for *x*: SS term). For SAXS intensity, CTR vs. CPL plots show a significant change in slope while for CTR-CHD, the CHD plot is significantly shifted with respect to CTR. Table reports analysis of data shown in Fig. 3, Fig. 4.

	Estimate	Std. Err.	t	F value	p	R^2^_adj_	p(SW)
**A**	**CTR vs CPL**						
**i) D period**	**D ∼ *x* + SS + *x*:SS (CTR vs. CPL)**						
(Intercept) [nm]	65.80	3.003e−02	2191.245	4.8016e + 06	< 2e−16***	0.6514	0.7981 (ns)
*x* [nm/%]	2.910e−03	5.288e−04	5.504	30.291	7.54e−07***		
SS	0.1046	3.003e−02	3.484	21.39	0.000912***		
*x*:SS	5.827e−04	5.288e−04	1.102	1.2141	0.274780 (ns)		

**ii) SAXS int.**	**Int ∼ *x* + SS + *x*:SS (CTR vs. CPL)**						
(Intercept) [a.u.]	170.863	22.939	7.449	55.4822	3.59e−10***	0.6399	0.6661 (ns)
*x* [a.u./%]	2.980	0.404	7.377	54.4228	4.77e−10***		
SS	18.279	22.939	0.797	0.6350	0.4286 (ns)		
*x*:SS	1.043	0.404	2.583	6.6712	0.0122*		

**B**	**CTR vs CHD**						
**i) D period**	**D ∼ *x* + SS + *x*:SS (CTR vs. CHD)**						
(Intercept) [nm]	65.84	2.748e−02	2395.655	5.739e + 06	< 2e−16***	0.5261	0.5574 (ns)
*x* [nm/%]	3.123e−03	4.798e−04	6.509	42.367	1.2e−08***		
SS	6.133e−02	2.748e−02	2.231	4.9792	0.0291*		
*x*:SS	3.703e−04	4.798e−04	0.772	0.596	0.4430 (ns)		

**ii) SAXS int.**	**Int ∼ *x* + SS + *x*:SS (CTR vs. CHD)**						
(Intercept) [a.u.]	132.771	22.838	5.814	33.798	1.94e−07***	0.6599	0.3339 (ns)
*x* [a.u./%]	3.497	0.399	8.771	76.922	1.11e−12***		
SS	56.371	22.838	2.468	6.093	0.0162*		
*x*:SS	0.527	0.399	1.322	1.747	0.1908 (ns)		

Further to the changes in D-period observed, there is also a marked difference in the measured intensity of the 5th order *I*(*q*) peak. Fig. 3b shows that at the surface of the tissue the intrafibrillar order (peak intensity) is similar in both the unloaded CTR and loaded CPL cases, but clear differences appear from around 30% tissue thickness onward. A reduction in intensity is observed in the CPL case, with the difference increasing to ∼50% reduction in intensity, and thus order toward the deepest zone. Linear model fits and ANOVA analysis show that (Table 1 A(ii)) a) there is an extremely significant (*p* < 0.001) change of intensity with *x* for CTR b) the initial intensity (at *x* = 0 in the superficial zone) is similar (*p* = 0.84), as seen from the overlap of the regression confidence intervals on the left and c) the slopes of the regression lines are significantly different (*p* < 0.001). This reduction in intensity complements the changes observed in the D-period, and taken together, implies a reduction in fibrillar ordering (given that compression will not change the total amount of collagen within the tissue and therefore cause intensity change). Indeed, if the degree of intrafibrillar ordering were constant pre- and post-compression, one would expect a 25% (from a compression of 20% ≡ 1/(1–0.2) = 125%) increase in intensity on loading rather than the observed decrease which therefore suggests a disordering effect. Finally, we note that although prior work has reported an increased strain within the superficial zone, as dictated by the strain profiles observed in full thickness cartilage [46], [47], the results in Fig. 3 cannot confirm or refute this, as due to interference from the loading platens we exclude the first (superficial) and last points in the scan from the regressions.

### 3.3 Effects of enzymatic degradation on the depth-wise fibrillar structure

**Fig. 4:**
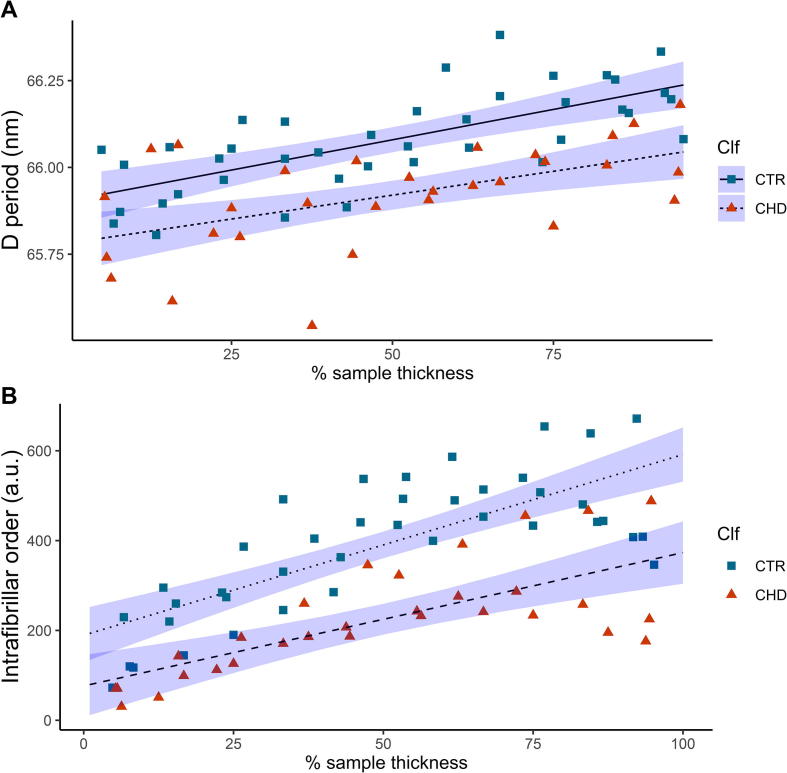
The effect of partial removal of the proteoglycan phase on the collagen D-period (a) and associated intrafibrillar order (b) in the absence of loading. Here the squares represent values prior to loading (Sample State (SS) = CTR, same data points as in Fig. 3) and the triangles represent post compression and relaxation (SS = CHD). Dotted (CTR) and dashed (CHD) lines denote results of the linear regression models: D ∼ X + SS + X: SS (for (a)) and Int ∼ X + SS + X:SS (for (b)). Shaded regions denote confidence intervals on the linear regressions. Linear regression and statistical parameters reported in Table 1.

Enzymatic digestion with chondroitinase ABC causes a reduction in the content of chondroitin sulphate, one of the side chains forming part of the proteoglycan molecules, and our previous study has shown that in the deep zone, the digestion causes a reduction in the fibrillar D-period as the compositionally driven swelling pressure is reduced [20]. Fig. 4a, comparing CTR and CHD groups in a similar manner to the CTRL/CPL comparison in Fig. 3a, shows that this effect can be observed throughout the thickness of the AC tissue and the effect is therefore not isolated to particular zones which are higher in proteoglycan content, such as the deep zone[11]. The changes in the D-period on chondroitinase treatment (Fig. 4a) are very similar to the change in D-period under loading (Fig. 3a). The qualitative impression of similar slopes but lowered intercepts is confirmed from the linear model fits and ANOVA results (Table 1 B(i)), where a) a significantly lowered (*p* < 0.05) D-period intercept (at *x* = 0) for CHD compared to CTR and b) no significant difference between the slopes (*p* = 0.443) is observed. This percentage difference remains around ∼0.2% throughout the thickness, which – presuming the proteoglycan reduction as the primary mechanism for the pre-strain change – suggests the alteration to the proteoglycan density to be consistent across the thickness, for the digestion protocol used. Further to this, there is also an associated reduction in the peak intensity (and therefore a reduction in intrafibrillar order), which increases on going into the deep zone (increasing *x*), as shown by Fig. 4b. ANOVA results show (Table 1 B(ii)) that there is a) a significant lowering of the CHD- regression line reflected in the significant (*p* < 0.05) value for the intercept (*x* = 0) term SS but b) a nonsignificant reduction in slope (*p* = 0.198 for the *x*:SS term) between the groups (in contrast to CTR-CPL). Both D-period and intrafibrillar order show (like CTR-CPL) extremely significant change with *x*. Lab-tests show that chondroitinase treatment led to significant sGAG release (Supplementary Information Experimental–Fig. S3) and significantly reduced mechanical properties (maximum tangent modulus; Supplementary Information Experimental–Fig. S4), as reported earlier [20].

## 4 Discussion

### 4.1 Depth dependent gradient in D-period and fibrillar ordering in native cartilage

**Fig. 5:**
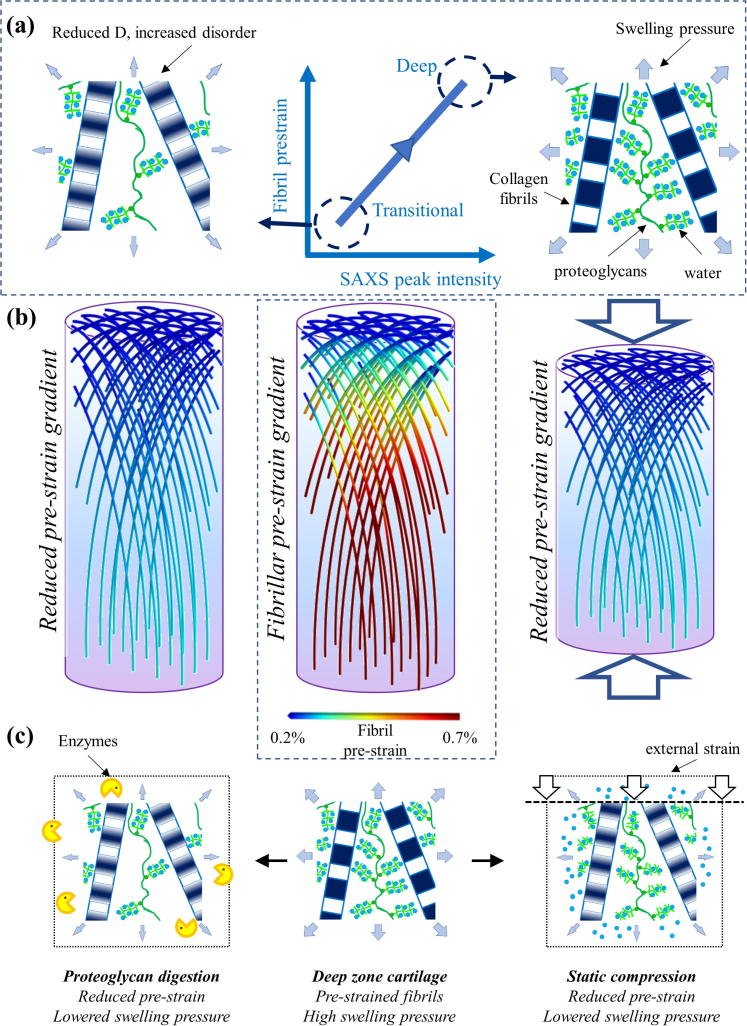
a) *Centre*: Positive correlation between fibrillar pre-strain and SAXS intensity (corresponding to ordered fibrils), with labels indicating tissue locations characterized by low and high values of these parameters. *Right*: Ultrastructure of cartilage ECM in the deep zone, showing a network of striated type II collagen fibrils confining a swollen, hydrated proteoglycan (PG) gel (green wavy lines: PGs; blue circles: water). *Left*: Ultrastructure in the transitional zone, with lower PG concentration leading to reduced swelling pressure. (b) *Centre*: Existence of a novel gradient of increasing fibrillar pre-strain from the surface/transitional (upper) zone to the deep (lower) zone in articular cartilage, from scanning SAXS measurements. The colour-scale on the fibrils indicate levels of pre-strain: blue – low pre-strain and red – high pre-strain (colour online) *Right*: Compression reduces the fibrillar pre-strain gradient (colour gradient reduces) in a similar manner to *Left*: treatment by chondroitinase sulphate to remove part of the PG phase. Note that the continuous fibre-representation in the schematics is meant only to indicate direction and prestrain levels of fibrils across the tissue depth, and not to imply individual fibrils spanning the superficial to deep zone range. (c) Ultrastructural mechanism enabling these fibrillar gradients: enzymatic (*left*) and load-induced (*right*) changes to the original fibrillar nanostructure (*centre*) result in loss of water molecules originally bound to PGs, thus reducing the internal pre-stress exerted on the fibrillar network by the PG-phase. (For interpretation of the references to colour in this figure legend, the reader is referred to the web version of this article.)

Cartilage possesses a complex structure that varies in both the microscale architecture and composition of the ECM components with depth (through the thickness of the tissue) and it is this structural gradient that is believed to allow cartilage to fulfil the combined roles of a frictionless articulating surface as well as a firm connection to subchondral bone [10], [17]. Here we have investigated whether, in a similar manner, depth-wise structural gradients of the nanoscale architecture of the collagen fibrils exist throughout the full thickness of cartilage. Our findings are summarized pictorially in Fig. 5, where the spatial gradient and nanostructural changes are shown in a combined manner. We first find that the D-period – a marker of fibrillar pre-strain – shows a clear depth dependent gradient, being lowest toward the superficial zone and in the transitional zone and largest in the deep zone. Note that due to the finite spatial resolution of the X-ray beam, possible cross-talk between adjacent tissue regions due to the relatively thick sample, and interference from loading platens at the start of the scan, we do not believe our current measurements can resolve the very thin uppermost superficial layer itself, which remains an area for future work. Nevertheless, we believe the increase in D period toward the deep zone is likely regulated by the changes in the local hydration – which is in turn controlled by the known depth-wise variation in proteoglycan content – which influences the swelling pressure induced onto the fibrils [48], [49], [50]. Previous studies, using an experimental protocol to determine the depth-dependent intrinsic fixed charge density, have shown that there is an increase with depth, which was used to infer a higher PG content as well [11]. This finding thus supports our observation that the deep zone fibrils express a larger D-period. Alongside the increase in D-period in the deeper zone, there is also an associated increase in the peak intensity and intrafibrillar order, and schematics of the fibrillar nanostructure in both cases are shown in Fig. 5a. SAXS peak intensity increases can arise from both i) an increased concentration of collagen (which is true of the deep zone [51]), as well as ii) an increase in the intrafibrillar regularity and ordering of the tropocollagen molecules [52] neglecting, for now, subtleties arising from different behaviours of different order SAXS peaks [20], [35]. Indeed, the two phenomena may be related: given that the increase in swelling pressure causes an increased pre-strain of the fibrils, this may therefore decrease the intra-fibrillar disorder that is associated with the fibrils being in a relaxed state [52]. This hitherto unknown gradient in fibrillar pre-strain and liquid crystalline ordering is shown schematically in Fig. 5b, centre, where the classical Benninghof arcade structure [15], [16] is enriched with a colorization of the fibrils, representing increasing levels of pre-strain from the surface to the deep zone found in our experiments. We note here that because we could not resolve clearly the thin superficial zone in all our samples, our findings of a gradient refer mainly to the transitional to deep zone. Future measurements with a smaller nanofocus X-ray beam may enable resolution of structure within the superficial layer, and possibly a higher pre-strain due to its confining role for the joint as a whole.

### 4.2 Compression leads to a disruption to the D-period and intrafibrillar order

Secondly, on compression of the tissue to physiological levels of 20%, here we show that there is an associated reduction in the D-period throughout the full thickness of the tissue, which is most prominent in the deep zone (Fig. 5b, right). To understand why this may occur, we note that it has been well documented [8], [49], [53] that on compression of cartilage, there is an outflow of the water (initially loosely associated with the PGs) from the interfibrillar space, which then would lead to a reduction in the overall hydrostatic pressure (Fig. 5c, right). This reduction in hydrostatic pressure in turn lowers the pre-stress on the surrounding stretched fibrillar network, leading to a relaxation of pre-strain, the (observed) reduction in D-period, and potentially a more disordered intrafibrillar arrangement of molecules associated with relaxed fibrils (as described above). Prior work using X-ray scattering considered change in fibrillar direction (and not D-period) and showed that under compressive mechanical loading, loading causes deep-zone fibrils to alter their orientation away from their initial direction (parallel to the load direction) [54]. Both of these mechanisms – increased fibrillar disorder as well as fibrillar rotation away from the loading direction – can help explain the observed reduction in peak intensity under compression. Increased intrafibrillar disordering will reduce peak intensities via a Debye-Waller mechanism at the molecular level [20], and rotation of fibrils away from the vertical will reduce the degree to which fibrils satisfy the diffraction (Ewald) condition in reciprocal space. However, to characterise the changes in peak intensity more precisely, a more challenging task will be to quantify changes in scattering length density (SLD) in both the extrafibrillar matrix and fibrils upon compression, due e.g. to loss of water from both compartments. Since water flow may occur both from the intra-fibrillar to extrafibrillar space, as well as from the extrafibrillar space to outside the tissue, which will alter the relative density differences between fibrils and extrafibrillar matrix in opposite ways, more sophisticated experimental designs incorporating, e.g., microfluid chambers with calibrated measures of water expulsion and absolute SAXS standards may provide insight into this in future.

### 4.3 Enzymatic digestion leads to a similar response to both depthwise D-period and intrafibrillar order changes

Thirdly, we find that tissue degradation in the form of enzymatic digestion using chondroitinase ABC has a clear effect on the established structural gradient of the fibrillar network. Whilst the trend of increasing D-period with depth remains, however, the D-period across all zones is smaller in the enzymatically treated cartilage than in that of the control cartilage, and the rate of increase is also reduced (Fig. 5b, left). When taken together with previous studies showing removal of the PG matrix leading to a reduction in the water content of the tissue [55], we can conclude therefore that the enzymatic treatment leads to a reduction in the hydrostatic pressure which, in turn, mediates a reduction in the fibrillar D-period (Fig. 5c, left). Interestingly, the magnitude of the reduction in fibrillar pre-strain across the zones is within the same range as observed under compression (Figs. 3 and 4), which suggests that the fibrils in enzymatically degraded cartilage – as found, e.g. from osteoarthritic or aged tissue or joints suffering from inflammation – are already in a relaxed state prior to any tissue level compression. This would have implications on how well cartilage is then able to respond to loading, as the enzymatically degraded ECM is already in a compromised state, and would not be able to resist further loading. We can speculate that such a mechanism may lead to abnormal mechanosignalling of the cells within the compromised or degraded cartilage ECM, as well as in the underlying bone, which could accelerate the mechanobiological feedback loop leading to increased cartilage wear and possible altered subchondral bone remodelling [25], [56]. As with case of the compressed cartilage, the SAXS peak intensity is significantly lower in enzymatically treated cartilage. As enzymatic removal of chondroitin sulphate does not remove the collagen phase (indeed, only partial removal of the PG phase itself occurs), this change in peak intensity can be directly correlated with a change in the intra-fibrillar organisation, specifically a more relaxed fibrillar network and associated intrafibrillar disordering. We note that the change in D-period and intrafibrillar order does not require complete removal of proteoglycan, and indeed it is possible that the enzymatic digestion is only partial. Further, the effect of the enzymatic treatment on intrafibrillar order shows up as a shift in the intercept, rather than a change in slope as observed for loaded tissue. While we do not have a clear explanation for this, factors such as the nature of the digestion protocol used (cores in media rather than intact joints) may play a role.

Previous studies have characterised the effect of selective matrix degradation on gross tissue mechanics [57], [58]. In particular, Rieppo et al. [58] use safranin-O staining of control and chondroitinase ABC treated cartilage samples to show significant decrease in proteoglycan concentration particularly in the superficial zone. It is therefore interesting to observe the spatial variation in chondroitinase induced changes in deep zone collagen organisation/nanoscale mechanics measured in the present study. This suggest that these collagen changes are dependent on both the loss of sGAG, induced by chondroitinase, and the inherent localised swelling pressure prior to digestion.

### 4.4 A pre-stressed fibril/matrix gel model of cartilage at the ultrastructural level

**Fig. 6:**
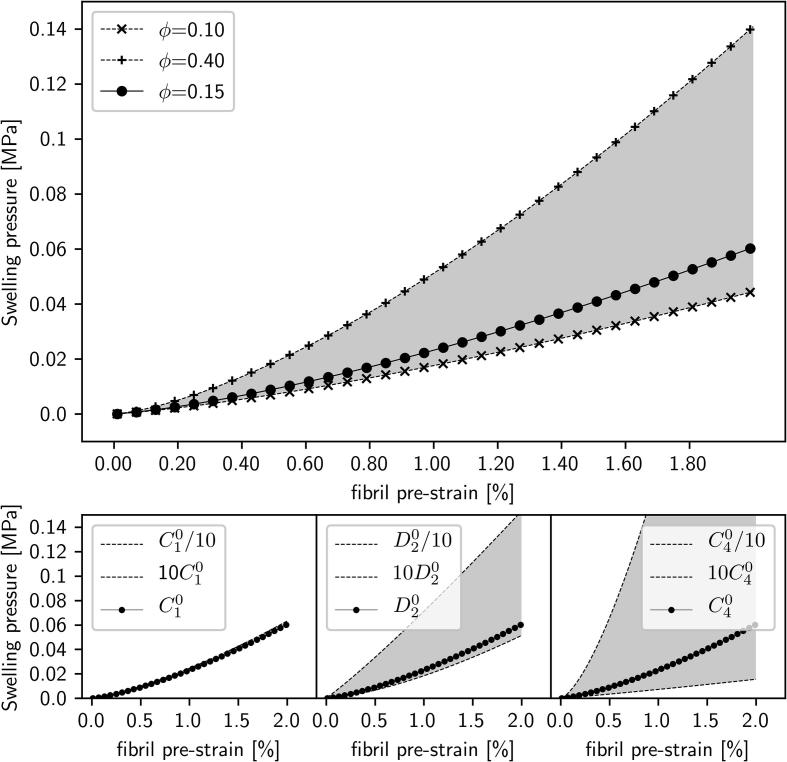
(a) Swelling pressure as a function of fibril volume fraction ϕ, showing an increase from 0 to about 0.05 MPa with fibril pre-strain increment of 2% (ϕ = 0.15; solid line, filled •s). Material parameters *C*_1_ = 4.34 × 10^3^ MPa, *D*_2_ = 2.7 × 10^4^ MPa and *C*_4_ = 2.0 MPa [26]. Upper (ϕ = 0.40; dashed lines, +s) and lower curves (ϕ = 0.10; dashed lines, ×s) show the range of variation of predicted swelling pressure on variation of the fibril volume-fraction ϕ. (b)–(d) Analogous variation of the material parameters *C*_1_, *D*_2_ and *C*_4_ (by a factor of 10 above and below the utilised values in Fig. 6(a)) show maximal sensitivity to variation in *C*_4_ (Fig. 6(d)) and least to variation in *C*_1_ (Fig. 6(b)).

These findings can be summarized in a simple model of the cartilage ultrastructure as a spatially-graded fibril-reinforced hydrogel (Fig. 5a, c and Fig. 6). We consider a representative volume element of the ECM at the micron/sub-micron scale in cartilage, containing mainly Type II collagen fibrils and hydrated proteoglycans (Fig. 5a). Highly ordered fibrils, with greater pre-strain (larger D) exist in the deep zone, and are shown by the schematic in Fig. 5a, left: regularly banded fibrils with a sharp gap/overlap interface, and high density of proteoglycans with bound water, exerting the composition driven swelling pressure (large arrows around the schematic) leading to pre-strain. The pre-strains on the collagen fibril leads to internal ordering of the liquid-crystalline arrangement of tropocollagen molecules inside the fibril (indicated by the sharp interface between gap (light) and overlap (dark) zones. Conversely, less ordered fibrils, with lower pre-strain (lower D) exist in the transitional zone, and are shown by the schematic in Fig. 5a, right. Here, the density of proteoglycans is reduced, leading to a lower swelling pressure (thinner arrows), and fibrils have both shorter D-period as well as a more diffuse gap/overlap ratio. We suggest these two features may be related – in an unstretched or relatively slack fibril, intrafibrillar molecular disordering, especially in the gap zone, will lead to a shortened gap-zone length and hence reduced total D-period. The difference between fibrils with differing degrees of pre-strain may be linked to the level of recruitment stretch referred to in [26], as well as to previous models of fibrillar structural alterations during tensile deformation in tendon [42]. Note that our introductory schematic in Fig. 2(h) showed these two effects – change in fibrillar strain and reduction in intrafibrillar order – separately, for clarity only, while here we refer to a combination of these effects.

Fig. 5c shows schematically the structural changes in the collagen/PG matrix under enzymatic treatment (left) and load (right). Enzymatic digestion shown schematically by enzymatic attack of MMP molecules like chondroitinase, reduce the number of proteoglycans relative to collagen, thus lowering the swelling pressure. On loading, the proteoglycans lose some of the bound water (blue circles), which reduces their degree of swelling or expansion (more crumpled conformation), thus reducing swelling pressure on the fibrils and leading to a similar reduction in pre-strain and increase in disorder as upon enzymatic digestion. Both effects reduce the swelling pressure on the fibrils – the first by a reduction in number of proteoglycan/water aggregates and the second by a reduction in number of bound water molecules, and reduce the pre-strain as well, indicated by the narrower outward arrows.

The physical picture described above can be quantified in the form of a simple structural/mechanical model of a pre-stressed fibrillar network with stiffness *C*_4_ inside a matrix of stiffness *C*_1_, in the presence of an additional pressure Π arising from the hydration-dependent swelling described above. The relation is given in Methods: Modelling, derived in Supplementary Information–Model (based on prior models [26], [27], [43], [44]), and the predictions are shown in Fig. 6a. It can be seen that the levels of swelling pressure changes due to relatively small (<1%) change in fibrillar pre-strain are of the order of 0–0.5 MPa, consistent with estimates of the swelling pressure in cartilage from prior modelling work on cartilage [26], [27], [59], [60] (as a reference, the typical peak maximum stress of cartilage in non-injurious loading is of the order of several MPa [7]). Hence the fibril pre-strain is an extremely sensitive probe of local stress-levels in the matrix, a concept which has been previously proposed by Masic et al. [61] based on controlled dehydration measurements of tendon collagen. Variation of the model parameters (range of variation between upper and lower control lines, shown as shaded area, in Fig. 6a–d) shows the model is most sensitive to changes in fibril volume fraction *ϕ*_f_ and C_4_, whilst relatively insensitive to the variation of *C*_1_ and *D*_2_. The model thus provides a link between the fibrillar pre-strain levels and the internal swelling pressure at the nanoscale, which is challenging to access via macroscopic measurements, and could potentially be used to diagnose alterations in local cartilage nanomechanics in inflammation, osteoarthritic degradation or other degenerative conditions.

Concerning limitations of our work, both technique- and biological-related aspects can be considered. We use 2D SAXS on an intrinsically 3D fibrillar architecture in cartilage, albeit controlling for this confounding factor by preparing samples with fibre symmetry axis along the vertical. Here the use of relatively thick samples (2 mm) compared to beam and step-size (∼20 μm) can – as illustrated in Fig. S1 in Supplementary Information – lead to crosstalk between adjacent regions (for beam tilt of <0.1°, of the order of 5 μm here; 1% sample thickness). Further, 3D effects such as load-induced out-of-plane tilting of fibrils – particularly in the deep zone with vertically oriented fibrils – may also occur, which cannot be captured by 2D SAXS measurements, and would also influence SAXS peak intensity. However, an estimate of this effect can be made, assuming fibre-symmetry through the sample, and estimating out-of-plane fibril tilting to be similar to the (small, <5°) in-plane tilting of the peak position in the 2D SAXS pattern under static compression case. With an ellipsoidal Gaussian intensity profile and using the typical angular width ∼20° of *I*(χ) (Fig. 1), the estimated intensity reduction from out-of-plane tilting would be (1 − exp(−½ (χ_1_/Δχ_0_)^2^)) ∼0.03 or 3%, which is much less than the ∼50% reduction seen in Fig. 3, Fig. 4. While we therefore do not believe out-of-plane tilting is playing an important role in this static compression case, SAXS tensor tomography [62], [63], [64] is a powerful new technique which can be used to resolve 3D fibril orientation in biocomposites, and in combination with *in situ* methods, may resolve these issues in the future especially for biocomposites where fibre orientation has more complex geometries than the quasi-1D vertical variation seen in the cylindrical samples used in the current study. Related to this, the analysis methodology used here could be refined to include angle-dependent variation of fibrillar parameters, to link to the 3D fibre-matrix models of cartilage developed previously [26], [27], [43], similar to our prior work in 3D diffraction modelling of cuticle [65]. Secondly, the biological matrix modification (partial removal of chondroitin sulphate) is a synthetic attempt to mimic the natural changes in proteoglycan composition that occur in ageing; in future, more biologically relevant analysis would include use of naturally aged tissue, together with the use of human cartilage – which can be analysed using SAXS as shown previously [20]. Thirdly, the number of samples is relatively small (due partly to the difficulty of accessing synchrotron to do these specialised experiments), and studies with larger sample numbers, possibly investigating intra- and inter-joint variations within a species, would be useful. Related to this, systematic pair-wise comparisons of tissue before and after loading (by using same sample numbers in CTR and CPL case) would be beneficial. Fourthly, the use of linear models for the variation of D-period and intrafibrillar order is an approximation; particularly for intrafibrillar order a nonlinear trend (downward) trend is visible for the data on the last quarter of the plots in Figs. 3b) and 4b). The use of either empirical nonlinear polynomial models to parametrize the trend, or better still, multiscale model-based predictions, would be preferable in future. Fifthly, the loading protocol used was static; cartilage is used in a dynamic mechanical environment, and future studies should investigate time-dependent and cyclic loading at the fibrillar level. Sixthly, the (small, <1–2%) fluctuations in primary beam intensity were neglected in comparison to the ∼40–50% changes in SAXS intensity under compression or enzymatic treatments. While we believe this is justified in our experimental configuration, we note that future experimental protocols dealing with potentially much smaller changes in SAXS intensity should correct for incident beam intensity fluctuations. Finally, although we have not tested cartilage from different load bearing regions or joints we suggest that the fundamental behaviour reported here is likely to be present in all articular cartilage although the precise magnitudes are likely to vary with the *in vivo* mechanical environment and associated gross mechanical properties of the tissue.

## 5 Conclusions

In summary, we demonstrate that there is a clear gradient in fibrillar pre-strain and intrafibrillar order through the thickness of articular cartilage, likely mediated by the localised changes in PG-derived hydration and associated swelling of the tissue. The measured gradient in the zonal fibrils are a combination of both an absolute change in the D-period as well as a disordering amongst the sub-units of the fibrils. Further, we show that under compression of cartilage, there is a reduction in the fibrillar pre-strain present within the ECM. Interestingly, this effect is closely mimicked by the response of the tissue to PG removal alone, highlighting both the importance of the internal hydration of the tissue, as well as the way in which nanoscale mechanical homeostasis in soft tissues can be disrupted by small variations in component matrix profiles. Our results thus provide both insight into understanding how age-related alterations in the matrisome of musculoskeletal, vascular and other collagen-rich tissues can significantly disrupt their biomechanical function, and may help develop, in future, avenues to detect and ameliorate such changes.
