# Supplementary material for: Proteoglycan degradation mimics static compression by altering the natural gradients in fibrillar organisation in cartilage
Source: Acta Biomater. 2019 Oct 1;97:437–50. doi: 10.1016/j.actbio.2019.07.055 (PMC6838783; doi:10.1016/j.actbio.2019.07.055)
Supplement: Supplementary data 1 [file mmc1.docx]

**Sample geometry and beam size:**


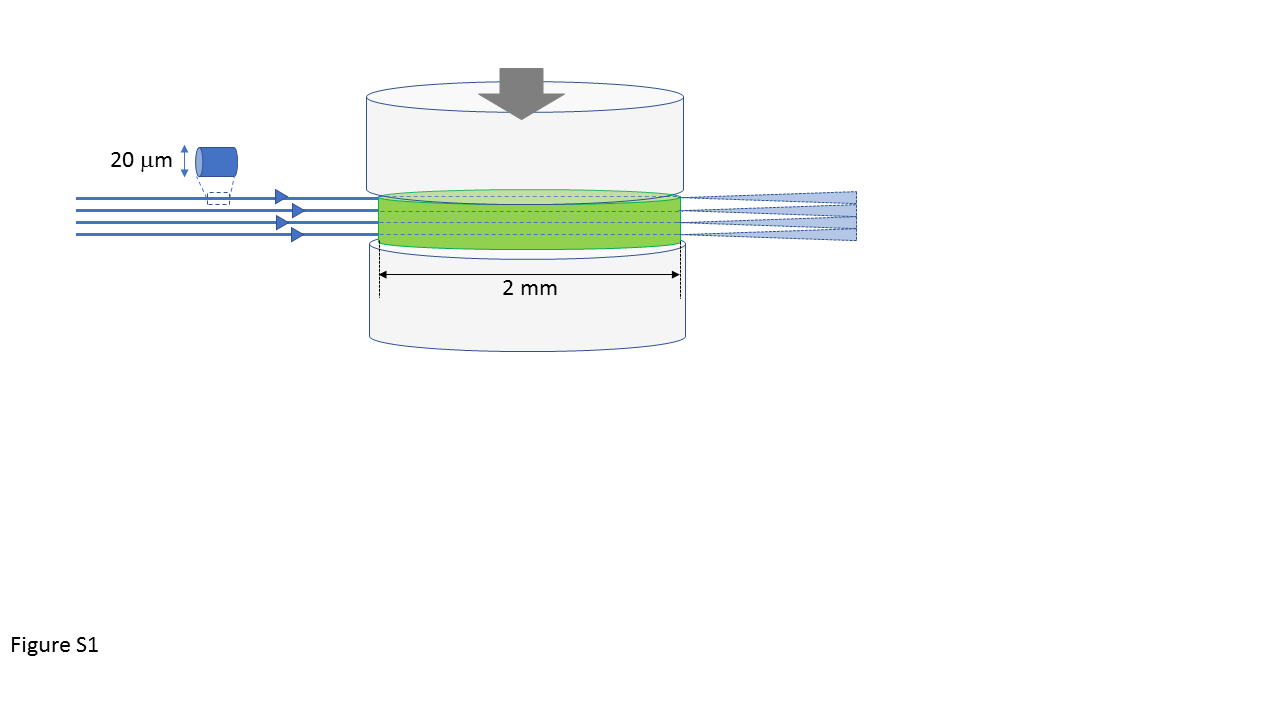


**Figure S1**: *Schematic of sample setup, showing issues involved in 2D scanning on 3D core of cartilage – volume (tunnel) effects, which can lead to cross-talk between adjacent steps for small beam tilts relative to the sample axis*

**Chondroitinase treatment and mechanical testing:**

The sGAG assay was used to measure the level of PG release following enzymatic digestion. The dye, 1,9-dimethylmethylene blue (DMB) complexes with sulphated GAG chains causing a metachromatic shift in the absorbance maximum from 600 to 535nm. The Ascent microplate reader is pre-programmed with a protocol and is able to detect such shifts. To begin with the DMB reagent was prepared using DMB, ethanol, sodium formate and formic acid (98/100%). Alongside this, a set of standards of chondroitin sulphate with fixed concentrations were prepared to act as reference values in determining sGAG levels using the same buffers as those used in the enzymatic digestions. A dilution series would later be used to form standard curves from which media concentrations could be back calculated from. The standard series used can be found in **Table S1**.

40µl of the standards were placed into a 96 well plate with 3 repeats. The media to be analysed were first vortexed and then 40µl transferred into each well (with 3 repeats per sample). These were then topped with 250µl of the DMMB reagent using a multichannel pipette. The plate was then placed into the micro-plate reader and read for absorbance at 595nm. A reduction in the absorbance directly correlates with an increase in the presence of sGAG. A mean absorbance value was calculated from the triplicates and the sGAG concentrations calculated from the standard curve, where an acceptable standard curve must have an R^2^ value above 0.99 as shown in **Figure S2**. Results showing the level of sGAG release are shown in **Figure S3**.

| Standard | Standard concentration (μg/ml) | Volume 100 µg/mL sGAG(μl) | Buffer Volume (μl) |
| --- | --- | --- | --- |
| STD0 | 0 | 0 | 1000 |
| STD1 | 5 | 50 | 950 |
| STD2 | 10 | 100 | 900 |
| STD3 | 20 | 200 | 800 |
| STD4 | 30 | 300 | 700 |
| STD5 | 40 | 400 | 600 |
| STD6 | 50 | 500 | 500 |
| STD7 | 60 | 600 | 400 |

**Table S1**: Table indicating standard concentrations and volume of both chondroitin sulphate and buffer used at each level.


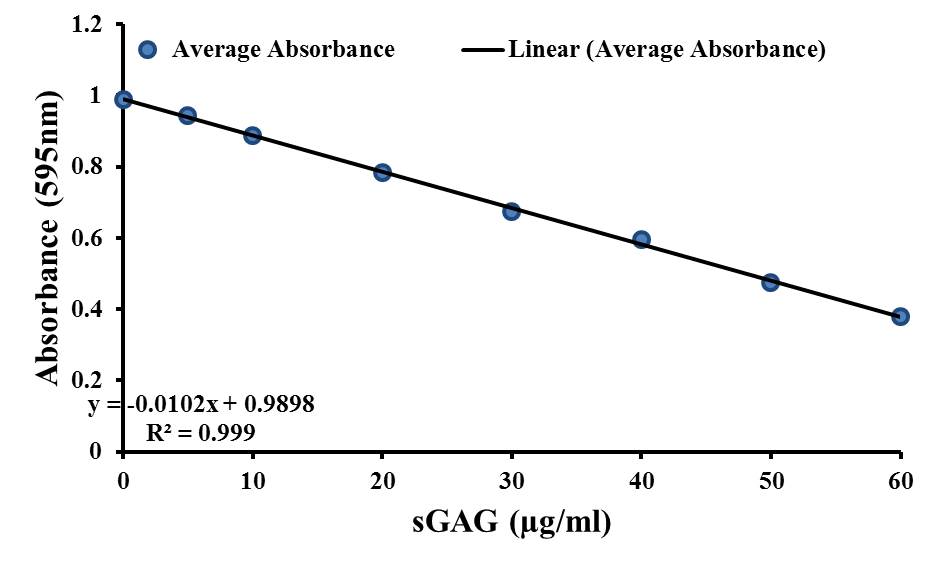


**Figure S2**: Graph showing a sample standard curve fitted with a linear regression with an R^2^ value of 0.999.

**Figure S3**: Chondroitinase ABC treatment leads to increased sGAG release in treatment media. Values represent mean values with error bars showing SEM for *n*=12/group (from lab tests).

**Figure S4**: Chondroitinase ABC treatment leads to reduced tissue stiffness. Tangent modulus is significantly reduced when explants are treated with Chondroitinase ABC at 0.1U/ml. Tangent modulus was calculated from the linear region of the stress-strain curve. Values represent mean values with error bars showing SD for n = 9/group.

**Radiation damage tests**:


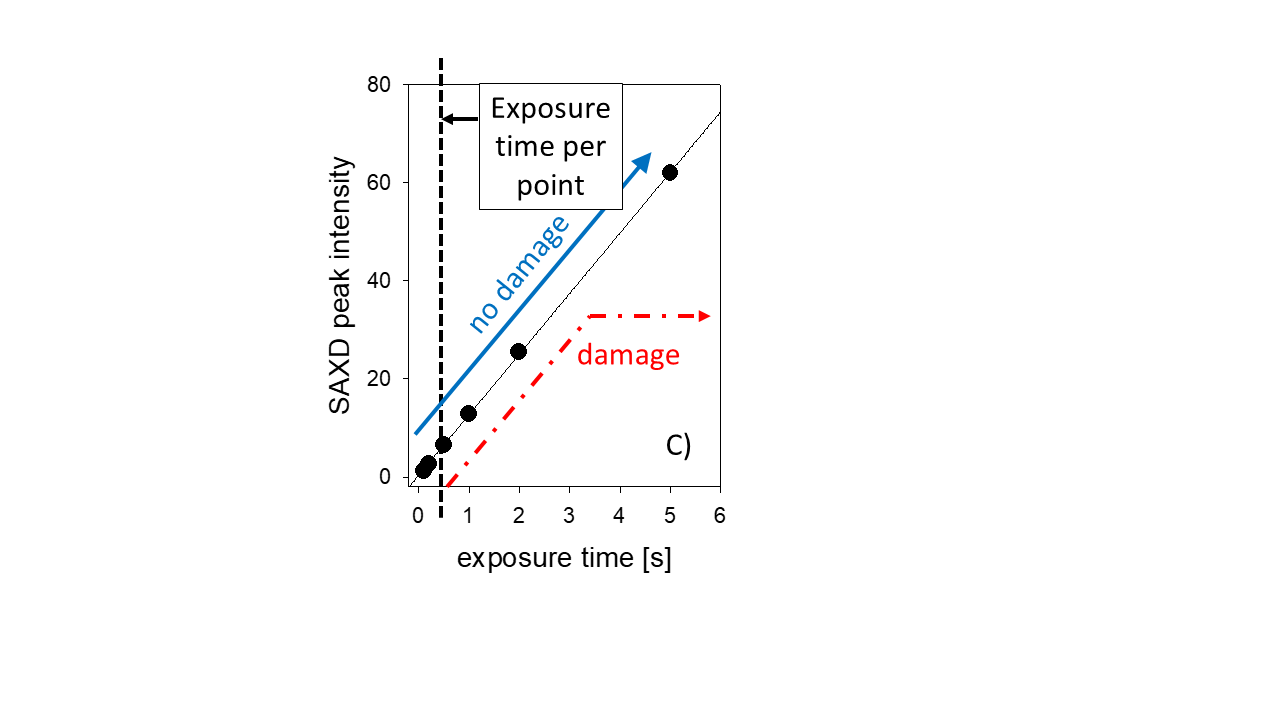


**Figure S5**: *Radiation damage tests*: Total SAXS peak intensity (after diffuse background subtraction) measured for 0.1, 0.5, 1, 2 and 5 second exposure times on bovine cartilage core (Each measurement at a point offset by > the beam diameter from the previous). The blue arrow indicates the expected trend if no radiation damage is perceptible over this time range. The red line indicates (one) hypothetical trend where damage has occurred from ~ 3 s, with a saturation of intensity with increasing exposure time indicating a reduced or vanished SAXS peak. The experimental points (solid line) follow the expected trend of no damage within the time interval shown. The vertical dashed line shows the exposure time used for the measurements reported in this work (0.5 s), which is well within the time interval (0 – 5 s) depicted.

**SAXS profiles:**


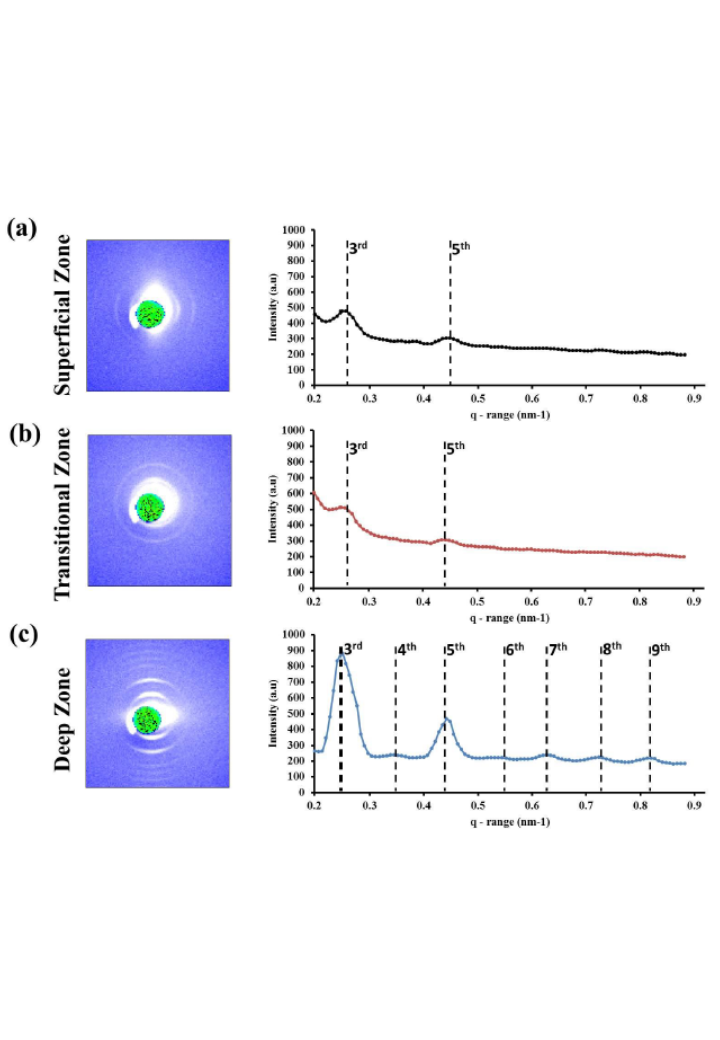


**Figure S6:** *Right*: *I*(*q*) profiles for the three SAXS patterns shown in Figure 2a) (reproduced on left), showing the greater SAXS intensity in the deep zone. Vertical dashed lines indicate approximate locations of different peak orders. *Left*: Corresponding 2D SAXS images.

**Figure S7**: Variation of incident beam intensity for a representative sample (from ionization chamber readings; arbitrary units) over the course of a SAXS scan, showing small fluctuations (1.6% of mean value in the example above).
