# Supplementary material for: Proteoglycan degradation mimics static compression by altering the natural gradients in fibrillar organisation in cartilage
Source: Acta Biomater. 2019 Oct 1;97:437–50. doi: 10.1016/j.actbio.2019.07.055 (PMC6838783; doi:10.1016/j.actbio.2019.07.055)
Supplement: Supplementary data 2 [file mmc2.pdf]

Proteoglycan degradation mimics static compression by altering the natural gradients in fibrillar organisation in cartilage - Supplementary Information

Sheetal R Inamdar[a], Ettore Barbieri[b], Nicholas J Terrill[c], Martin M Knight[a], Himadri S Gupta[a,*]

[a] *Institute of Bioengineering and School of Engineering and Materials Science*
Queen Mary University of London
Mile End Road, E1 4NS, London, UK
[b] *Japan Agency for Marine-Earth Science and Technology (JAMSTEC)*
Department of Mathematical Science and Advanced Technology (MAT)
Yokohama Institute for Earth Sciences (YES)
3173-25, Showa-machi, Kanazawa-ku, Yokohama-city, Kanagawa, 236-0001, Japan
[c] *Harwell Science and Innovation Campus*
Diamond Light Source, Harwell, Didcot, OX11 0DE, United Kingdom

Abstract

We use the equations of mechanical equilibrium to derive an expression for the swelling pressure in hydrated cartilage. We consider a structural model composed of a pre-stressed fibrillar network inside a matrix.

Keywords: swelling pressure, Neo-Hookean, finite deformation, sensitivity analysis

1. The swelling pressure

At equilibrium in free swelling conditions, the Cauchy (true) stress $\boldsymbol{\sigma}$ in the tissue is zero:

$$\boldsymbol{\sigma} = -\Delta\Pi\,\mathbf{I} + (1-\phi_F)\,\boldsymbol{\sigma}_M + \phi_F\,\boldsymbol{\sigma}_F = 0 \tag{1}$$

where $\Delta\Pi$ is the swelling pressure, $\mathbf{I}$ is the identity tensor, ϕ_F is the volume fraction of the fibrillar network, $\boldsymbol{\sigma}_M$ is the true matrix stress, $\boldsymbol{\sigma}_F$ is the true fibre stress. From equation (1), we get

$$\Delta\Pi = \frac{1}{3}\,\mathrm{trace}\Big((1-\phi_F)\,\boldsymbol{\sigma}_M + \phi_F\,\boldsymbol{\sigma}_F\Big) \tag{2}$$

The constitutive model of the matrix is assumed isotropic Neo-Hookean, while the one for the fibrils is anisotropic Neo-Hookean. The strain energy density function W_M for the Neo-Hookean model is (Holzapfel, 2001)

$$W_M = C_1\,(I_1 - \log I_3 - 3) + D_2\,(\log I_3)^2 \tag{3}$$

where $C_1 = \mu/2$ with μ being the shear modulus, $D_2 = K/2$ with K being the bulk modulus, $I_1 = \mathrm{trace}(\mathbf{C})$ and I_3 are respectively the first and the third invariant of the *Right Cauchy-Green* strain tensor $\mathbf{C}$

$$\mathbf{C} = \mathbf{F}^T\mathbf{F} \tag{4}$$

* Corresponding author
Email address: h.gupta@qmul.ac.uk (Himadri S Gupta)

where $\mathbf{F}$ is the *deformation gradient*, and $I_3 = J^2$, where $J = \det \mathbf{F}$.

The strain energy density function W_F for the anisotropic Neo-Hookean model is (Ateshian et al., 2009; Nagel and Kelly, 2013)

$$W_F = C_4 \, (I_4 - 1)^{\beta} \tag{5}$$

where C_4 and β are material parameters and I_4 is the invariant

$$I_4 = \mathbf{a}_0^T \, \mathbf{C} \, \mathbf{a}_0 \tag{6}$$

where $\mathbf{a}_0$ indicates the direction of the fibrils (Holzapfel, 2001), with Cartesian components

$$\mathbf{a}_0 = \begin{bmatrix} \sin\varphi \, \cos\theta \\ \sin\varphi \, \sin\theta \\ \cos\varphi \end{bmatrix} \tag{7}$$

with φ being the polar angle and θ the azimuthal angle. The value of β follows the restriction $\beta > 2$, which allows a smooth transition for the fibril response from compression to tension (Ateshian et al., 2009).

The *Second Piola-Kirchhoff* stresses are given by

$$\mathbf{S}_M = 2 \, \frac{\partial W_M}{\partial \mathbf{C}} \qquad \mathbf{S}_F = \int_{-\pi}^{\pi} \mathrm{d}\varphi \int_0^{2\pi} 2 \, \frac{\partial W_F}{\partial \mathbf{C}} \, \sin\varphi \; \mathrm{d}\theta \tag{8}$$

where the anisotropic fibrillar stress has been averaged over all the possible directions. The *Second Piola-Kirchhoff* stress is related to the Cauchy stress through

$$\sigma = \frac{1}{J} \, \mathbf{F} \, \mathbf{S} \, \mathbf{F}^T \tag{9}$$

Let us assume an isotropic deformation

$$\mathbf{F} = \lambda \, \mathbf{I} \tag{10}$$

with λ being the stretch ratio, $\lambda = 1 + \epsilon$ with ϵ being the strain. Then, substituting equation (9) into equation (2) leads to (Nagel and Kelly, 2013)

$$\Delta\Pi = (1 - \phi_F) \, \frac{2}{\lambda^3} \left[C_1 \left(\lambda^2 - 1 \right) + 12 \, D_2 \, \log\lambda \right] + \phi_F \, \frac{8}{3} \, \pi \, C_4 \, \beta \, \frac{(\lambda^2 - 1)^{\beta - 1}}{\lambda} \tag{11}$$

References

Ateshian, G.A., Rajan, V., Chahine, N.O., Canal, C.E., Hung, C.T., 2009. Modeling the matrix of articular cartilage using a continuous fiber angular distribution predicts many observed phenomena. Journal of biomechanical engineering 131, 061003.

Holzapfel, G.A., 2001. Nonlinear solid mechanics: a continuum approach for engineering science. John Wiley & Sons, Chichester, New York.

Nagel, T., Kelly, D.J., 2013. Altering the swelling pressures within in vitro engineered cartilage is predicted to modulate the configuration of the collagen network and hence improve tissue mechanical properties. Journal of the mechanical behavior of biomedical materials 22, 22–29.
